# Geocoding accuracy and the recovery of relationships between environmental exposures and health

**DOI:** 10.1186/1476-072X-7-13

**Published:** 2008-04-03

**Authors:** Soumya Mazumdar, Gerard Rushton, Brian J Smith, Dale L Zimmerman, Kelley J Donham

**Affiliations:** 1 Department of Geography, University of Iowa, Iowa City, IA, USA; 2 Department of Biostatistics, University of Iowa, Iowa City, IA, USA; 3 Department of Statistics and Actuarial Science and Department of Biostatistics, University of Iowa, Iowa City, IA, USA; 4 Department of Occupational and Environmental Health, University of Iowa, Iowa City, IA, USA

## Abstract

**Background:**

This research develops methods for determining the effect of geocoding quality on relationships between environmental exposures and health. The likelihood of detecting an existing relationship – statistical power – between measures of environmental exposures and health depends not only on the strength of the relationship but also on the level of positional accuracy and completeness of the geocodes from which the measures of environmental exposure are made. This paper summarizes the results of simulation studies conducted to examine the impact of inaccuracies of geocoded addresses generated by three types of geocoding processes: a) addresses located on orthophoto maps, b) addresses matched to TIGER files (U.S Census or their derivative street files); and, c) addresses from E-911 geocodes (developed by local authorities for emergency dispatch purposes).

**Results:**

The simulated odds of disease using exposures modelled from the highest quality geocodes could be sufficiently recovered using other, more commonly used, geocoding processes such as TIGER and E-911; however, the strength of the odds relationship between disease exposures modelled at geocodes generally declined with decreasing geocoding accuracy.

**Conclusion:**

Although these specific results cannot be generalized to new situations, the methods used to determine the sensitivity of results can be used in new situations. Estimated measures of positional accuracy must be used in the interpretation of results of analyses that investigate relationships between health outcomes and exposures measured at residential locations. Analyses similar to those employed in this paper can be used to validate interpretation of results from empirical analyses that use geocoded locations with estimated measures of positional accuracy.

## Background

Geocodes are geographic references for computer records that lack them [1]. In environmental health research, geocodes provide geographical references for people and environmental contaminants. While traditional environmental health research is often aspatial, it is becoming increasingly common to address environmental epidemiological questions through spatial analyses using finely geocoded data [1]. Attempts to establish relationships between environmental exposures and health depend on the accuracy of the geocodes. When health outcomes are sensitive to the magnitude of the exposures in question, any loss of accuracy can cause a loss in the ability to establish relationships between the two. Geocoding quality has become an issue in epidemiological and environmental health studies [1-15]. Different studies use different criteria to judge the quality of geocodes [1], although two measures of quality are widely recognized: positional accuracy and completeness in ascertaining a geocode for a given address. In most studies, the severity of these problems is related to the process that generates the geocodes.

The motivating question for the research in this paper is, how do errors in geocodes affect estimates of the relationship between environmental exposures and health outcomes? Statistical power in a model measuring the relationship between exposures and health is computed for different geocoding processes. The results are intended to help researchers decide whether a geocoding method under consideration in an environmental health study is adequate for risk assessment. A second motivating question asks whether it is possible to know the level of geocoding accuracy that is needed to establish the health risk of environmental contaminants in an area. We assume that the contaminant locations can be measured precisely and that the locations of persons exposed to the contaminants are subject to uncertainty. Our approach is similar to that taken by Rull and Ritz [16], who measured the loss in relationships between exposure and health outcomes due to exposure misclassification. We focus on a particular and common cause of exposure misclassification – the geometric inaccuracy of the geocodes. In this research, we analyze the positional inaccuracy of rural geocodes. There is evidence to show that rural geocodes are susceptible to larger inaccuracies than urban geocodes [4,10]. Geocoding inaccuracies are therefore a more pressing problem with rural geocoding than urban ones, although the method described in this paper can be easily adapted to urban situations.

## Methods

### Overview

We use an experimental method to determine the effect of geocoding inaccuracy on the ability to recover relationships between environmental exposures and health. In our experiments, hypothetical risk models are used to simulate health outcomes for a given spatial pattern of environmental contaminants and a given spatial pattern of exposed individuals. For the given spatial pattern of contaminants, we generate health data for hypothetical individuals living at known address locations in Carroll County, Iowa. The address locations used to calculate the environmental contaminant values and subsequently generate the expected health outcomes are highly accurate geographic locations obtained through geocoding the residential structures corresponding to each address based on their recognition on a properly registered, orthophoto map. This geocoding process is abbreviated as G^o^. We then ask how this known relationship compares with estimated relationships between environmental exposures and health outcomes based on two other methods for geocoding the addresses. One method uses the emergency responders geocoding process – G^E ^(E-911 geocoding) – and the other uses the well known automated address-matching approach using TIGER line files from the US census. (G^T^, with and without offset). TIGER is an acronym for Topologically Integrated Geographic Encoding and Referencing. In the experiments described in more detail below, measures of exposures are degraded because of geocoding errors in the locations of individuals. The effect of these errors is assessed by examining the accuracy of resulting odds ratio estimates. It is not our objective in this study to determine which geocoding process is optimal. Such an analysis could be a natural extension of this work. In this study we develop methods to study the effect of geocoding inaccuracy on the relationships between environmental exposure and health. We realize this using the three exemplar geocoding processes. In the next section, we discuss the theoretical framework underlying our approach.

### Theoretical Framework

While it is possible to apply the method outlined in this research to aggregated health/environmental data (e.g. aggregated at the level of the Census tract), we confine this discussion to the use of individual level address data. We assume that the dataset consists of N unique addresses, with one individual resident at each address. Like Armstrong et al [17], we let the N×5 matrix **X = [I, A, W, Z, L] **denote the environmental epidemiological data, where **I**_N×1 _is a vector of unique identifiers for each record and **A**_N×1 _is a vector of corresponding addresses. The vector **W**_N×1 _provides the health statuses for individuals, **Z**_N×1 _gives the environmental exposures, either measured or modelled, and, and **L**_N×P _contains other covariate information where **P **possible covariates are available. Following our earlier definition [1] therefore, a geocoding process G is used to assign geographic coordinates (U_i_, V_i_) to the i*th *address, so that G(A_i_) = (U_i_, V_i_). Different geocoding processes could yield different coordinates for the same address. However, since measured/modelled exposures are assumed known at every location, we can define a GIS (Geographical Information Systems) model 'm' that maps coordinates (U_i_, V_i_) to exposure Z_i_; i.e. m(U_i_, V_i_) = Z_i_. Hence, we can see that the contaminant value is a function of the geocoding process G since

m(G (A_i_)) = Z_i_.

In addition, the expected health effect E(W_i_) can be modelled as a function of Z_i _and covariates L_i _as E(W_i_) = g(Z_i_, L_i_), where g( ) is often a linear or logistic regression model. A simpler approach is to model health outcomes as a function of the environmental contaminant only; i.e.

E(W_i_) = g(Z_i_). It can thus be seen that the model relating W to the contaminant is a function of the geocoding process as well:

E(W_i_) = g (m (G (A_i_))).

Note that given a function 'g', a GIS contaminant model 'm', and known A_i_' s, the left hand side of equation (2) can be simulated from the right hand side. W_i _can often be represented by a binary variable. For example, in population-based studies cases could be coded as 1s and controls as 0s. Alternatively, if the study design is a proportionate incidence or proportionate mortality study, then a certain ICD-9 (International Classification of Diseases-Version 9) code can be coded as 1 and all other ICD-9 codes as 0. In such instances, we can express W_i _as a Bernoulli random variable where:

P(W_i _= 1) = *π*_i_

P(W_i _= 0)= 1-*π*_i_

and *π*_i _is the probability of developing disease condition W_i_.

The probability function of W_i _is:

$$\text{f}\left({\text{W}}_{\text{i}}\right)=\pi_{\text{i}}^{{\text{W}}_{\text{i}}}1-\pi_{\text{i}}^{{\text{W}}_{\text{i}}},{\text{W}}_{\text{i}}=0,1.$$

The relationship between *π*_i_, Z_i_, and W_i _is usually modelled using the logistic function as:

$$\text{E}\left({\text{W}}_{\text{i}}\right)=\pi_{\text{i}}=\frac{{\text{e}}^{\beta_{0}+\beta_{1}{\text{Z}}_{\text{i}}}}{1+{\text{e}}^{\beta_{0}+\beta_{1}{\text{Z}}_{\text{i}}}}$$

The contaminant values Z are continuous in nature, and the associated model parameter is interpreted as follows: every unit increase in exposure to the contaminant Z causes an increase of ${\text{e}}^{\beta_{1}}$ in odds of disease. The quantity ${\text{e}}^{\beta_{0}}$ is interpreted as the prevalence of disease among unexposed (Z = 0) individuals.

From equations 1 and 4, we can write:

$$\text{E}\left({\text{W}}_{\text{i}}\right)=\pi_{\text{i}}=\frac{{\text{e}}^{\beta_{0}+\beta_{1}\text{m}(\text{G}({\text{A}}_{\text{i}}))}}{1+{\text{e}}^{\beta_{0}+\beta_{1}\text{m}(\text{G}({\text{A}}_{\text{i}}))}}$$

From 5, we see that for a given address, relationship ${\text{e}}^{\beta_{1}}$ base prevalence ${\text{e}}^{\beta_{0}}$ and GIS model m, the probability of disease for a person varies as a function of the geocoding process G. Conversely, if by some means the exact probability of disease for a person at address A_i _were known, then the disease odds for exposure, or *β*_1_, would vary from one geocoding process to another. If the exact probabilities were calculated using a gold standard or exact geocoding process G, then the extent to which the odds ratio of ${\text{e}}^{\beta_{1}}$ and the corresponding odds ratio from another geocoding process G' agree would reflect the quality of the geocoding process G'. This odds ratio can thus be used as a means of exploring the quality of one geocoding process with respect to another.

With reference to equation (4), under the null hypothesis, there is no relationship between exposure Z and health outcomes. The odds of disease from having been exposed is therefore 1.

$${\text{H}}_{0}:\beta_{1}=0\;{\text{or equivalently e}}^{\beta_{1}}=1$$

Under the non-informative alternative hypothesis, the odds of disease is different from zero. While an exposure to contamination usually increases the odds of disease and we can expect this to be greater than one, we allow for the possibility of the odds being less than one; i.e. an alternative hypothesis of

$${\text{H}}_{\text{A}}:\beta_{1}\neq 0\;{\text{or equivalently e}}^{\beta_{1}}\neq 1$$

For a given alternative $\beta_{1}^{*}$ sample size N, and Type-I error probability *α*, there is a direct relationship between statistical power 1 – B (where B is Type-II error) and the variance of Z. Again from equation 5 (and 1), if everything other than the geocoding process is kept fixed then power varies with the type of geocoding used. If the 'exact probabilities' were calculated using a 'gold standard' or 'exact' geocoding process G and any other geocoding process were used to detect the relationship *β*_1 _then power would vary from one geocoding process to another. Equations are available [18] for calculating sample size or power in the situation where Z is t or normally distributed. Unfortunately, environmental contaminants are rarely found to be distributed normally. As an alternative, simulation methods can be used to ascertain power. A simple procedure is followed in this paper:

a) Disease data W are simulated according to a known relationship g(.) between Z and W.

b) All model parameters other than G remain constant, and an effort is made to estimate the relationship between Z and W. The extent to which the estimated relationship varies from the true relationship, as G varies, is a measure of the decline in the quality of G. In this study, we apply this procedure to a real situation occurring in Carroll County, Iowa. The three types of geocoding processes examined are typical of those that are used for counties in the Midwestern U.S.

### Address Geocoding

The data we wish to develop consist of residential sites and associated contaminant values. Three geocoding processes were used to develop these datasets:

#### a) Address-matching using TIGER line files (G^T^)

These are geocodes in which addresses are matched to Census street centerline files. Centerline files are produced by the U.S. Census and were available to us from the E.S.R.I's (Environmental Systems Research Institute) website [19]. For this research, Census 2000 TIGER line files are used. Addresses were matched to the street centerline files using the GIS package ArcGIS 9.1 [20]. TIGER geocodes are placed by the software on the centreline by interpolating location on the basis of the street address. End offset of 3% and side offsets in feet (meters) of 0, 200(60.96), 400(121.92), 600(182.88) and 800(243.84) were applied to the TIGER geocodes. Throughout this paper we refer to the TIGER geocoding process G^T ^as one process which includes geocoding with and without offsets.

#### b) E-911 geocoding (G^E^)

E-911 geocodes are a promising means of accurately geocoding rural addresses [21]. For the purpose of emergency services dispatch, all discrete addresses are geocoded so that they may be located in response to a 911 telephone call requesting assistance. In geocoding addresses in this Iowa County, this location was defined as that which would most enable an emergency responder to find the person who had requested the service. Specifically, the location is the geographic coordinates at which the emergency responder would leave the public road and join the private road leading up to the property from which the call was made. These geocodes were obtained as a GIS layer file from the Carroll County G.I.S coordinator. The data are current as of June 2006. No offsets are used with the E-911 geocodes.

#### c) Orthophoto map-based geocoding (G^o^)

**Figure 1:**
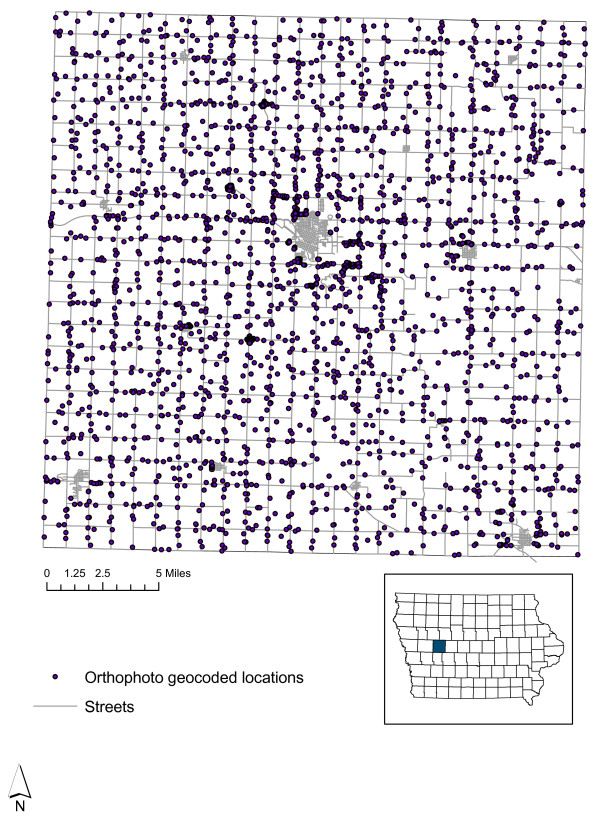
Locations of the geocoded rural addresses in Carroll County, Iowa.

Using visual identification, the E-911 rural addresses were 'enhanced' to a location centered on the residence location related to the address. This task was accomplished with the aid of 6 inch (15.2 cm)/pixel and two feet (61 cm) per pixel orthophoto maps of the study area, current as of 2002. Figure 1 displays the locations of the geocoded addresses over Carroll County. This dataset was provided by the Carroll County GIS office. A GIS data layer indicating the parcel to which a particular property belonged (and which is used by the county assessor's office for tax assessment) was overlaid on the Orthophoto map and E-911 address layers, to confirm that the geocode was being assigned to the correct address in the few cases when visual identification could not unambiguously identify the E-911 rural address with the related property.

Parcel geocoding was not considered as a reliable geocoding method in these analyses. The median parcel size for the properties of both farm and non-farm residences in rural Carroll county is 1,618,703 square feet or 179,856 yards (150,382 square meters), so that a geocode placed at the centre of a square parcel of this size would have a median error of approximately 671 feet (204 m). This error can be reduced with the help of ancillary knowledge of the location of the residence. Since the likely source of this knowledge would be an orthophoto image, anyone possessing this source would be better advised to extract the location of the residence as we have done in this work.

**Figure 2:**
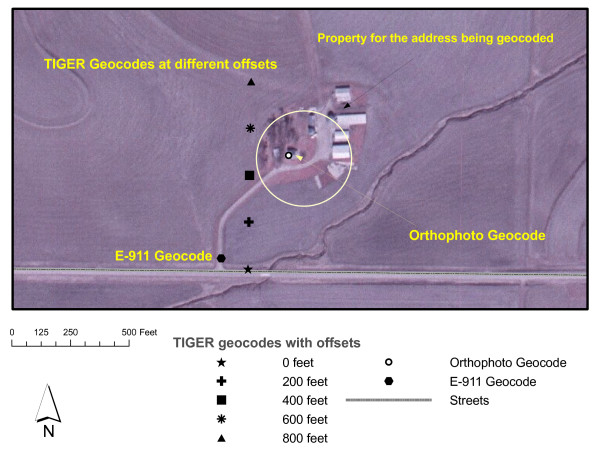
Illustration of three types of geocoding : orthophoto, E-911 and TIGER with offset, for the address 10392 260^th ^Street.

Figure 2 illustrates these geocodes. It shows the seven locations that we consider for addresses. The geocode on the public road leading to the property is the E-911 location and the geocode on the residence is the orthophoto geocode. In this case, the two geocodes are approximately 550 feet (168 m) apart. The TIGER geocodes, of which there are five, have varying degrees of accuracy in this example, with some of the TIGER offset geocodes having better accuracy than E-911.

We started with a comprehensive dataset of 2,516 addresses representing all rural addresses in Carroll County. All addresses that are located outside the legal (incorporated) boundaries of towns are considered rural. For each address an E-911 geocode is available. The E-911 geocodes therefore have 100% completeness. Since the orthophoto geocodes are enhanced from the E-911 geocodes, all addresses have an orthophoto geocode. Of the 2,516 addresses 14 were found to be duplicates and eliminated. A further 69 addresses were found to be have been erroneously coded as rural and removed. The remaining 2,443 addresses were geocoded to TIGER street centerline files. A minimum match score of 100 % was used and no manual interactive matching was used because the purpose of this research is to show the effects of typical differences in locations between "perfectly geocoded" residences according to currently accepted geocoding processes (automated TIGER, E-911) and ground truth as exemplified by the orthophoto determined locations. 1, 581 of the 2,443 addresses were geocoded with 100% match score to the TIGER Street Centerline files indicating a match rate of 64.7%. Our results represent a conservative view of the difference between TIGER, E-911 geocoded locations and ground-truth locations. Clearly, addresses that could not be geocoded accurately from the TIGER file would represent a systematically larger error than those studied here and bias would be introduced by any attempt to interactively geocode the unmatched addresses.

This research thus utilizes the 'incomplete' [22] set of 1,581 addresses. Therefore for each of these 1,581 addresses three geocodes – E-911, Orthophoto and TIGER are available. The next step is calculating contaminant values (Z). This is calculated using these geocodes and a GIS model 'm'.

### Contaminant value calculation

In this research we utilize CAFOs (Concentrated Animal Feeding Operations) as the disease-causing contaminant source. CAFOs have been suspected as possible sources of disease-causing effluents in rural areas of the U.S. [23,24]. Exposure to air from swine CAFOs has been suspected to increase the risk of eye irritation, headaches, nausea and a variety of respiratory and gastrointestinal disorders [23,25,26]. CAFO air is considered to hold elevated levels of H_2_S, Ammonia and suspended particles. Very few studies have attempted to look at the health effect of CAFOs making them an interesting source of pollution to study. In this study we attempt to work with the relationship between these contaminants and asthma. The study can be generalized to any other respiratory disorder like asthma that has an odds elevation and disease base prevalence similar to the ones assumed in this study, and that has a suspected relationship with one or more of the contaminants.

The locations of 55 CAFOs in Carroll County, for which permits had been issued by the state were obtained as a GIS layer file. A plume dispersal model based on the AERMOD (AMS/EPA Regulatory Model) [27] was used to model the contaminant dispersed from each CAFO. The contaminant modelled is a generic "conservative" contaminant which means that the contaminant is non reactive in nature. Our model can therefore apply to any and all of H_2_S, Ammonia and suspended particles. The model is a Gaussian dispersal model which accounted for prevailing wind direction. The input variables to the model are the wind direction, speed and the height of the stack. Meteorological data, averaged over five years, are from the National Weather Service Station at Sioux Falls; while the height of the stack is approximated at 5 meters. This was used to determine time-averaged (five years) relative concentrations of an air contaminant dispersed from a CAFO. The relative contribution of each CAFO is proportional to the size of the CAFO measured in number of animal units. The sum of the estimated emission values from all CAFOs was computed for each of the geocoded addresses. It is important to note that the contaminant values are relative and do not have any units in absolute terms. Thus, for example, a contaminant value of 300 at a residence implies that the contaminant there is 300 times the least possible contaminant value. For all N addresses, we thus have m(G^o ^(A)) = Z^o^, m(G^T ^(A)) = Z^T ^and m(G^E ^(A)) = Z^E^.

The model was realized with a combination of MaTLab [28] and Excel VBA (Visual Basic for Applications) [29] programs. The MaTLab program calculates the plume from a single CAFO and outputs the result as a 25 meter fine grid (over a 1 kilometre square CAFO pollution plume footprint) as a digital file. The contaminant value at each grid point is provided in the digital file. The Excel VBA program uses this digital file plume output and the locations of CAFOs and geocoded addresses to calculate the contaminant value at each address location. This program can calculate the contaminant value at any location in the County, be it an address location or any other chosen location. This table of contaminant values at each geocoded address is the input data for the simulation step discussed in the Simulation section below.

**Figure 3:**
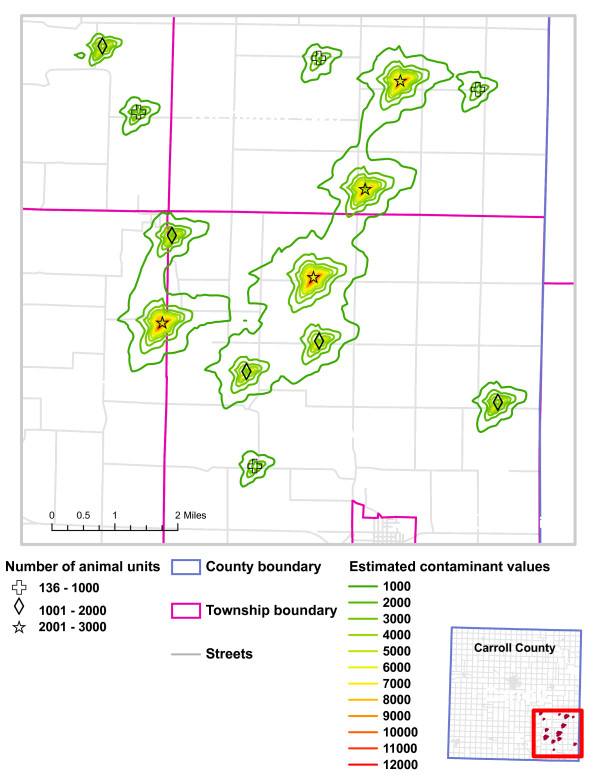
Estimated values of CAFO emissions in a region of South-East Carroll County, Iowa.

For the purposes of visualization, contaminant values were also computed for a 50 meter fine grid and the values were contoured in ArcGIS [20] to produce a surface representation. A small part of the resulting map is shown in Figure 3. In the next section we discuss the computer simulation which generates the modelled relationships and tests their strength in the presence of geocoding error. The simulation was performed using the R statistical software on a standard Pentium desktop.

### Simulation

The simulation methodology consists of the following 8 steps:

1) Assume that one individual resides at each address. Simulate probabilities of disease for N = 1,581 individuals – *π*_N×1 _using equation (5) but replacing G(A_i_) with G^o^(A_i_), a specific geocoding process, as:

$$\text{E}\left({\text{W}}_{\text{i}}\right)=\pi_{\text{i}}=\frac{{\text{e}}^{\beta_{0}+\beta_{1}\text{m}({\text{G}}^{\text{o}}({\text{A}}_{\text{i}}))}}{1+{\text{e}}^{\beta_{0}+\beta_{1}\text{m}({\text{G}}^{\text{o}}({\text{A}}_{\text{i}}))}}$$

We take *β*_0 _= ln (0.075). This implies that the simulated prevalence of disease among unexposed individuals is 7.5%. Further take *β*_1 _= ln (1.2)/(Interdecile(Z)). This implies that a person at the 90^th ^percentile of the contaminant distribution Z has an odds of 1.2 compared to a person at the 10^th ^percentile of the contaminant distribution. The 7.5% disease prevalence is consistent with reported population estimates for asthma, and the exposure odds ratio value of 1.20 is consistent with available risk estimates [30-32].

2) Randomly sample M individuals out of N. (See step 7 for values of M)

3) For a given sample generate disease outcomes according to the distribution W_i _~ binomial (*π*_i_,1) (i = 1 to M)

4) Our objective here is to recreate the relationship that was used in simulating the health data in step 1. In trying to do this, we consider (i) that we only have a sample of M addresses/people from our dataset of N people/addresses and (ii) that the contaminant values given to us have been calculated using all three forms of geocoding which are m(G (A^o^)) = Z^o ^(Orthophoto geocoding), m(G (A^T^)) = Z^T ^(TIGER geocoding) and m(G (A^E^)) = Z^E ^(E-911 geocoding). For any given person/address, different logistic regression estimates of $\beta_{1}({\hat{\beta }}_{1}^{\text{O}},{\hat{\beta }}_{1}^{\text{E}},{\hat{\beta }}_{1}^{\text{T}})$ and $\beta_{0}({\hat{\beta }}_{0}^{\text{O}},{\hat{\beta }}_{0}^{\text{E}},{\hat{\beta }}_{0}^{\text{T}})$ are obtained from the models (a), (b) and (c) as shown below.

$$\begin{array}{ll} \text{a}) & \text{E}({\text{W}}_{\text{i}})=\pi_{\text{i}}=\frac{{\text{e}}^{\beta_{0}^{\text{O}}+\beta_{1}^{\text{O}}\text{m}({\text{G}}^{\text{o}}({\text{A}}_{\text{i}}))}}{1+{\text{e}}^{\beta_{0}^{\text{O}}+\beta_{1}^{\text{O}}\text{m}({\text{G}}^{\text{o}}({\text{A}}_{\text{i}}))}}\\ \text{b}) & \text{E}({\text{W}}_{\text{i}})=\pi_{\text{i}}=\frac{{\text{e}}^{\beta_{0}^{\text{E}}+\beta_{1}^{\text{E}}\text{m}({\text{G}}^{\text{E}}({\text{A}}_{\text{i}}))}}{1+{\text{e}}^{\beta_{0}^{\text{E}}+\beta_{1}^{\text{E}}\text{m}({\text{G}}^{\text{E}}({\text{A}}_{\text{i}}))}}\\ \text{c}) & \text{E}({\text{W}}_{\text{i}})=\pi_{\text{i}}=\frac{{\text{e}}^{\beta_{0}^{\text{T}}+\beta_{1}^{\text{T}}\text{m}({\text{G}}^{\text{T}}({\text{A}}_{\text{i}}))}}{1+{\text{e}}^{\beta_{0}^{\text{T}}+\beta_{1}^{\text{T}}\text{m}({\text{G}}^{\text{T}}({\text{A}}_{\text{i}}))}} \end{array}$$

Thus, for a given person/address, while the outcome is the same for all the models, the predictor in a) is m(G (A^o^)) = Z^o ^; in b) it is m(G (A^E^)) = Z^E ^; and in c) it is m(G (A^T^)) = Z^T^.

5) Repeat steps 2 – 4 10,000 times.

Estimates of ${\hat{\beta }}_{1}^{\text{O}},{\hat{\beta }}_{1}^{\text{E}},{\hat{\beta }}_{1}^{\text{T}}$ obtained over the 10,000 simulations are averaged. Let us call these averaged estimates B^O^, B^E^, B^T ^respectively. Note that the TIGER geocoding process was used with five different offsets, therefore five separate models were run for model (c) as $\text{E}\left({\text{W}}_{\text{i}}\right)=\pi_{\text{i}}=\frac{{\text{e}}^{\beta_{0}^{\text{T}0}+\beta_{1}^{\text{T}0}\text{m}({\text{G}}^{\text{T}0}({\text{A}}_{\text{i}}))}}{{\text{1+e}}^{\beta_{0}^{\text{T}0}+\beta_{1}^{\text{T}0}\text{m}({\text{G}}^{\text{T}0}({\text{A}}_{\text{i}}))}}$ for 0 offset, $\text{E}\left({\text{W}}_{\text{i}}\right)=\pi_{\text{i}}=\frac{{\text{e}}^{\beta_{0}^{\text{T20}0}+\beta_{1}^{\text{T}0}\text{m}({\text{G}}^{\text{T20}0}({\text{A}}_{\text{i}}))}}{{\text{1+e}}^{\beta_{0}^{\text{T20}0}+\beta_{1}^{\text{T20}0}\text{m}({\text{G}}^{\text{T20}0}({\text{A}}_{\text{i}}))}}$ for 200 feet offset etc. There are also separate estimate values ${\hat{\beta }}_{1}^{\text{T}0},{\hat{\beta }}_{1}^{\text{T}200}$ etc for each offset.

6) Recovered odds (or the recovered relationship) are calculated as: ${\text{e}}^{{\text{B}}^{\text{O}}/\Delta }$, ${\text{e}}^{{\text{B}}^{\text{E}}/\Delta }$ and ${\text{e}}^{{\text{B}}^{\text{T}}/\Delta }$; where Δ = interdecile(Z^o^). Power (or the probability of detecting the relationship) is calculated as:

Power = (Number of significant ${\hat{\beta }}^{\#}$)/10,000, where # = O, E, T.

Finally, Confidence Intervals are calculated as:

${\text{e}}^{{\text{B}}^{\#}/\Delta }$ ± 1.96 * Standard Error {${\text{e}}^{{\text{B}}^{\#}/\Delta }$}

7) Steps 2 to 6 are repeated with different values of M, to study how power varies with sample size, for different geocoding processes. The values of M chosen were 316, 474, 632, 790, 948, 1106, 1264, 1422 and 1581 which correspond to 20%, 30%, 40%, 50%, 60%, 70%, 80%, 90% and 100% of N respectively.

Sensitivity analysis was carried out to test the behaviour of the simulation when subjected to different values of input parameters. Specifically the ${\text{e}}^{\text{Interdecile}(\text{Z})*\beta_{1}}$ value was taken to be 1.01, 1.15, 1.2 and 2.0. The effects of these input values on the average odds detected and the power were studied. The full sample (1,581) people was used for the analysis.

## Results

**Figure 4:**
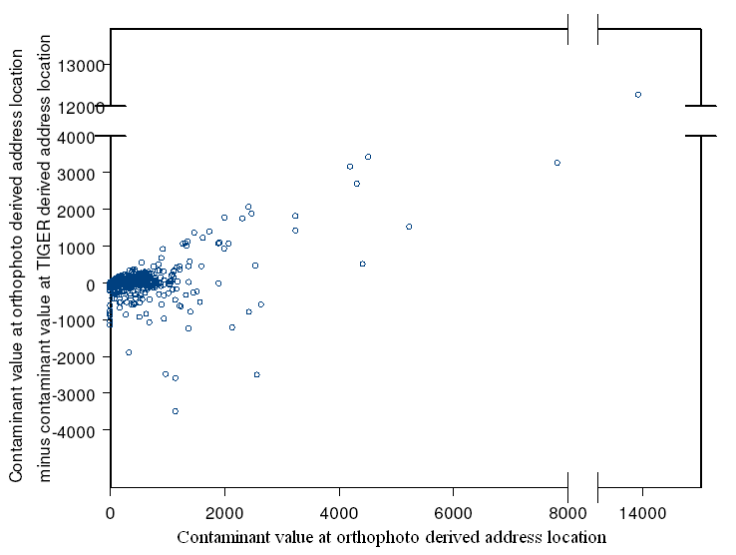
Relationship between error in contaminant values at TIGER geocodes with true contaminant value.

**Figure 5:**
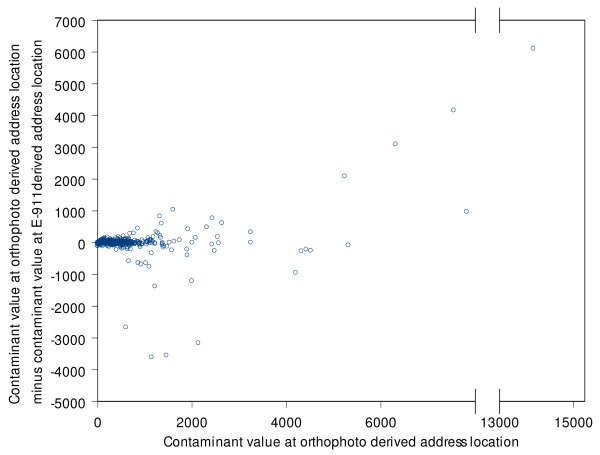
Relationship between error in contaminant values at E-911 geocodes with true contaminant value.

We define a geocoding error as the difference in distance units between the Orthophoto geocode and the geocode (E-911, TIGER) for a given address. Analyses of the TIGER (G^T^) geocoding errors and the E-911 geocode errors showed a median difference of 693 feet (211.23 m) for TIGER geocodes and 151 feet (46 m) for E-911 geocodes. Table 1 summarizes the errors between the orthophoto geocodes and other geocodes. Note that median error seems to be minimized at around 400 feet (122 m) offset for the TIGER geocodes. The largest errors with TIGER geocoding are in the range of 8 miles, which is caused by addresses in one part of the county being wrongly matched to a TIGER line file in another part of the county. These matching errors can be contrasted with the more frequent, but smaller offset errors. These errors (which are perhaps represented by the median) are in the range of 600 feet (183 m) for the TIGER geocodes. Earlier studies with a similar dataset have shown that if the outliers (represented by match errors) are removed then these errors can fit some well known distributions [3]. Contaminant values at E-911 geocoded locations and orthophoto map-based locations of addresses were highly correlated (Table 2). Figures 4 and 5 display the variation in errors with contaminant values. Note that while both E-911 (Figure 4) and TIGER (Figure 5) 19 geocoding have larger errors with increasing contaminant values, errors at smaller values seem to be more pronounced with TIGER geocoding. The outliers are addresses that are erroneously geocoded closer to the CAFOs than their true location. In fact these figures demonstrate that TIGER geocoding errors tend to introduce a pronounced positive bias in the contaminant values at address locations.

**Table 1:** Summary statistics for distance errors.

**TIGER Geocoding**	**Summary Statistics (In feet)**					
**Offsets (feet)**	**90^**th **^Percentile**	**75^**th **^Percentile**	**Mean**	**Median**	**Min**	**Max**

**0**	3702.83	2137.26	1631.09	692.87	20.82	46600.53
**200**	3634.15	2058.53	1556.22	654.15	3.97	46400.56
**400**	3641.91	2040.28	1582.01	615.19	20.70	46401.50
**600**	3658.40	2055.12	1653.10	670.92	38.85	46403.30
**800**	3687.35	2095.46	1751.19	814.97	27.68	46405.97

**E-911 Geocoding**	698.36	318.25	281.07	150.92	6.32	3196.67

**Table 2:** Correlations between contaminant values for each of 1,581 rural addresses for seven different geocoding methods.

**Geocoding Type**		**Orthophoto**	**E-911**	**TIGER geocoding with offset in feet**				
				**0**	**200**	**400**	**600**	**800**
**Orthophoto**		1.00						
**E-911**		0.90	1.00					
**TIGER geocoding with offsets in feet.**	**0**	0.65	0.75	1.00				
	**200**	0.68	0.79	0.96	1.00			
	**400**	0.71	0.79	0.94	0.97	1.00		
	**600**	0.70	0.80	0.93	0.94	0.97	1.00	
	**800**	0.71	0.81	0.89	0.92	0.93	0.96	1.00

**Figure 6:**
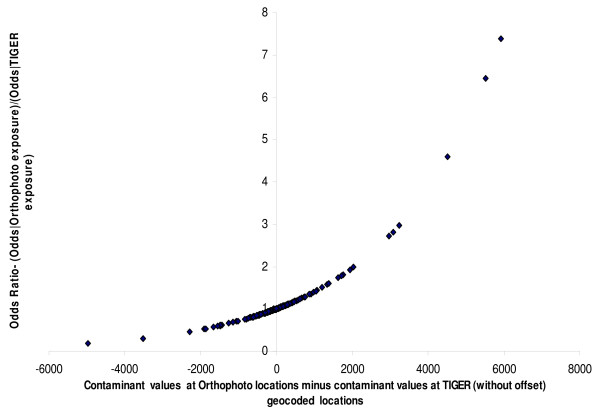
Variation in odds ratios in simulated disease from exposure to contaminant calculated to Orthophoto geocode and exposure to contaminant calculated to TIGER geocode, with error in contaminant calculation at a TIGER geocode.

**Figure 7:**
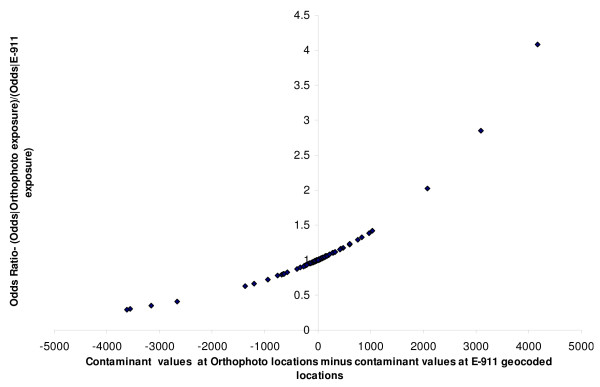
Variation in odds ratios in simulated disease from exposure to contaminant calculated to Orthophoto geocode and exposure to contaminant calculated to E-911 geocode, with error in contaminant calculation at an E-911 geocode.

One exploratory method of comparing the effect of errors in contaminant values from geocoding errors is the method of calculating the attenuation of odds ratios [16] The odds of disease at a geocoded location for an address can be calculated as a function of the contaminant value as for example ${\text{e}}^{(1.2/\Delta )*{\text{Z}}^{\text{O}}}$, where Z^O ^is the contaminant value calculated using the orthophoto geocode for an address and Δ is the interdecile range (Z^o^). Similarly the odds value of ${\text{e}}^{(1.2/\Delta )*{\text{Z}}^{\text{E}}}$ would represent the odds calculated using the E-911 geocode. Z^O ^- Z^E ^would represent the bias or error in calculating the contaminant value and this bias would in turn affect the odds ratio ${\text{e}}^{(1.2/\Delta )*{\text{Z}}^{\text{O}}}$/${\text{e}}^{(1.2/\Delta )*{\text{Z}}^{\text{E}}}$. The bias introduced by the error in contaminant values from geocoding inaccuracies could cause the odds of disease to be both greater or less than what we would expect it to be if the geocodes were accurate and there were no modelling error in the contaminant values. The ratio of odds calculated in the no error situation to that calculated with error would be 1 if this error were equal to zero, or so small that the ratio is equal to 1 when rounded to two significant decimal digits. To study the extent of the bias, odds ratios were calculated as odds (disease | Z^O^)/odds (disease | Z^E^) and odds (disease | Z^O^)/odds (disease | Z^T^). The results can be seen in Figures 6 and 7. In either of these figures, the more unbiased a given geocoding process is, the more we would expect the data points to cluster at odds ratio (OR) = 1. A larger proportion of the odds were unbiased (OR = 1) with E-911 geocoding (80.00%) than with TIGER geocoding with 0 offset (59.00%). Adding offset to the TIGER geocodes did not substantially improve the proportion of unbiased odds, with the mean being around 60%. There was also a bias towards detecting an association (OR < 1) with TIGER geocodes (≈20%), than with E-911 geocodes (10%). This is consistent with the observations made earlier from Figures 4 and 5.

We tested the robustness of the simulation by changing the value of the simulated odds. The results of this sensitivity analysis are summarized in Table 3 and Table 4. This was done for the 100% sample of 1581 addresses locations. Different values of simulated odds do not cause large differences in bias. The simulation program was tested with odds values of 1.01, 1.15, 1.2, 1.5 and 2.0 (with 1.2 being the value used in our main analyses). All these odds were recovered with reasonable accuracy by the geocoding processes (Table 3). However, as we might expect varying the odds value does have an effect on power (Table 4). An odds of 1.01 is successfully detected in only around 5% of the simulations by the various geocoding processes. In contrast an odds of 2.00 is detected with a power of 100%.

**Table 3:** Simulated mean estimates across fixed values of the true odds and the geocoding method.

**Geocoding Type**		**True values of the odds**				
		
		**1.01**	**1.15**	**1.2**	**1.5**	**2**
**Orthophoto**		0.98	1.15	1.21	1.51	2.02
**E-911**		0.99	1.16	1.21	1.44	1.76
**TIGER geocoding with offsets in feet.**	**0**	0.99	1.15	1.21	1.45	1.74
	**200**	0.99	1.15	1.21	1.47	1.80
	**400**	0.99	1.19	1.25	1.56	1.91
	**600**	0.99	1.17	1.24	1.50	1.82
	**800**	1.00	1.19	1.26	1.54	1.87

**Table 4:** Simulated power estimates across fixed values of the true odds and geocoding method.

**Geocoding Type**		**True values of the odds**				
		
		**1.01**	**1.15**	**1.2**	**1.5**	**2**
**Orthophoto**		5.6	74.9	93.2	100.0	100
**E-911**		5.0	65.7	87.0	100.0	100
**TIGER geocoding with offsets in feet.**	**0**	4.9	37.3	57.5	99.8	100
	**200**	5.2	40.7	62.1	99.9	100
	**400**	5.1	44.5	66.1	99.9	100
	**600**	4.8	43.7	65.9	99.9	100
	**800**	5.0	45.5	67.5	100.0	100

**Figure 8:**
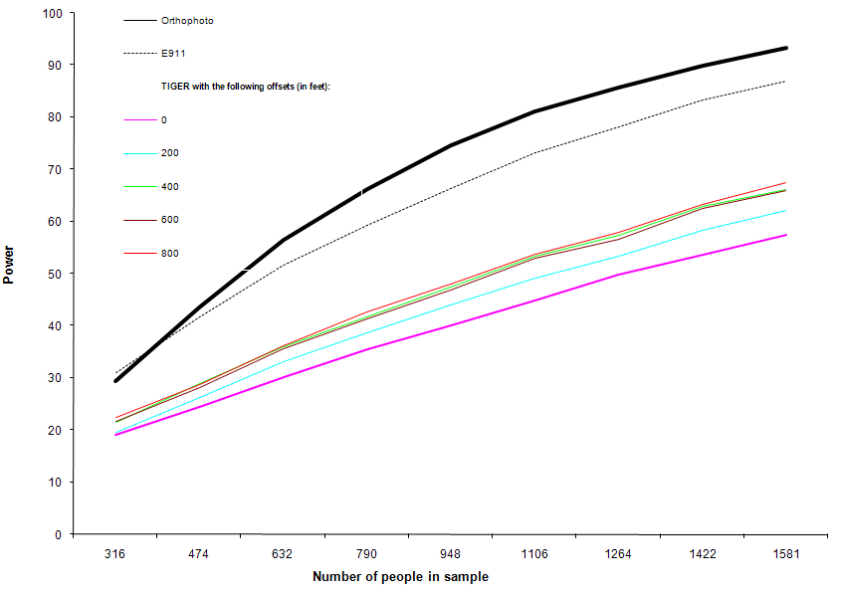
Estimated power as a function of sample size and geocoding method.

The relationships (odds) are recovered with almost no error across different sample sizes and geocoding processes (Table 5). The power is greater when E-911 geocodes were used than when TIGER geocodes were used, for a given sample size. TIGER geocoding provides very low power for the most part and it needs more than twice the sample size as that of E-911 or orthophoto geocoding to achieve the same power (Figure 8). The biased contaminant values (Figures 4, 5, 6, 7) at the TIGER geocodes contribute to this result. Table 6 compares odds recovered with varying TIGER offsets. Note that adding offset to the TIGER geocodes does increase power. The best power is obtained by using offsets in feet (meters) of 400 (121.92), 600(182.88) or 800(243.84) and the differences in power between the three are small. This can partly be explained by the fact that the median TIGER error is around 700 feet (213 m). It is therefore possible, that the 'optimal' TIGER offset is around this value. The higher offsets also result in an odds ratio which is slightly biased towards the greater than the true odds ratio. This could be because adding offset to the TIGER geocodes moves the address locations closer to the CAFOs, which are almost always located at an offset from the main street.

**Table 5:** Recovered odds ratios (true value is 1.2) by types of geocoding and number of people in a sample. Estimates and power are based on 10,000 simulated samples.

**Number of people in sample**	**Geocoding Type**	**Estimates**		**Power (%)**
			
		**Mean Odds Ratio**	**95%CI**	
**316**	**Orthophoto**	1.18	0.52–1.71	29.4
	**E-911**	1.17	0.53–1.65	31.1
	**TIGER with 0 offset**	1.18	0.50–1.82	19.0
**474**	**Orthophoto**	1.19	0.75–1.56	43.4
	**E-911**	1.19	0.75–1.52	41.8
	**TIGER with 0 offset**	1.19	0.69–1.66	24.3
**632**	**Orthophoto**	1.20	0.87–1.49	56.5
	**E-911**	1.20	0.87–1.45	51.8
	**TIGER with 0 offset**	1.20	0.78–1.56	30.2
**790**	**Orthophoto**	1.20	0.95–1.44	66.1
	**E-911**	1.20	0.94–1.42	59.4
	**TIGER with 0 offset**	1.20	0.86–1.51	35.5
**948**	**Orthophoto**	1.21	0.99–1.42	74.5
	**E-911**	1.21	0.98–1.39	66.4
	**TIGER with 0 offset**	1.20	0.90–1.48	40.1
**1106**	**Orthophoto**	1.21	1.04–1.40	81.1
	**E-911**	1.21	1.02–1.38	73.3
	**TIGER with 0 offset**	1.21	0.94–1.46	44.9
**1264**	**Orthophoto**	1.20	1.05–1.38	85.6
	**E-911**	1.21	1.04–1.36	78.3
	**TIGER with 0 offset**	1.20	0.95–1.43	49.7
**1422**	**Orthophoto**	1.21	1.07–1.37	89.7
	**E-911**	1.21	1.06–1.35	83.5
	**TIGER with 0 offset**	1.21	0.98–1.41	53.6
**1581**	**Orthophoto**	1.21	1.08–1.36	93.2
	**E-911**	1.21	1.08–1.35	87.0
	**TIGER with 0 offset**	1.21	1.00–1.40	57.0

**Table 6:** Recovered odds ratios by TIGER geocoding with variable offsets and number of people in a sample. Estimates and power are based on 10,000 simulated samples.

**Number of people in sample**	**TIGER geocoding with the following offsets in feet.**	**Estimates**		**Power (%)**
			
		**Mean Odds Ratio**	**95%CI**	
**316**	**0**	1.18	0.50–1.82	19.0
	**200**	1.18	0.50–1.84	19.6
	**400**	1.22	0.50–1.91	21.4
	**600**	1.20	0.50–1.84	21.7
	**800**	1.23	0.49–1.91	22.4
**474**	**0**	1.19	0.69–1.66	24.3
	**200**	1.19	0.68–1.67	26.3
	**400**	1.24	0.68–1.74	28.9
	**600**	1.22	0.69–1.68	28.3
	**800**	1.24	0.68–1.74	28.8
**632**	**0**	1.20	0.78–1.56	30.2
	**200**	1.20	0.79–1.58	33.1
	**400**	1.24	0.79–1.66	35.9
	**600**	1.22	0.80–1.60	35.6
	**800**	1.25	0.79–1.65	36.2
**790**	**0**	1.20	0.86–1.51	35.5
	**200**	1.20	0.86–1.52	38.7
	**400**	1.25	0.86–1.60	41.7
	**600**	1.23	0.87–1.55	41.2
	**800**	1.25	0.86–1.60	42.7
**948**	**0**	1.20	0.90–1.48	40.1
	**200**	1.21	0.90–1.49	44.0
	**400**	1.25	0.92–1.56	47.5
	**600**	1.23	0.92–1.51	46.9
	**800**	1.25	0.92–1.56	48.1
**1106**	**0**	1.21	0.94–1.46	44.9
	**200**	1.21	0.94–1.46	49.1
	**400**	1.25	0.96–1.54	53.2
	**600**	1.23	0.96–1.49	52.9
	**800**	1.26	0.96–1.54	53.8
**1264**	**0**	1.20	0.95–1.43	49.7
	**200**	1.21	0.96–1.44	53.4
	**400**	1.25	0.97–1.51	57.3
	**600**	1.23	0.97–1.47	56.6
	**800**	1.25	0.98–1.51	58.0
**1422**	**0**	1.21	0.98–1.41	53.6
	**200**	1.21	0.98–1.42	58.3
	**400**	1.25	1.00–1.49	62.8
	**600**	1.23	1.00–1.45	62.4
	**800**	1.26	1.01–1.49	63.4
**1581**	**0**	1.21	1.00–1.40	57.5
	**200**	1.21	1.00–1.41	62.1
	**400**	1.25	1.02–1.48	66.1
	**600**	1.24	1.02–1.44	65.9
	**800**	1.26	1.03–1.48	67.5

## Discussion

This paper investigated the degree to which the recovery of a known relationship between environmental exposure and health is affected by the geocoding quality of the subjects of the research. Power analyses showed that the quality associated with different geocoding processes affected the ability to recover the relationships. As with all power analyses the size of the sample as well as the variability in the contaminant surface and the location of the sample in relation to this surface also affected the ability to recover the relationship. Because state or local regulations often control the locations of CAFOs relative to the residences of people, the numbers of people living in areas of high exposure to CAFO contaminants is limited which, in turn, limits the ability to detect health effects in natural experiments as in this research [33]. Another limitation of this study is the spatial structure of the contamination surface. The structure of the surface we use is limited to the source of pollutants and the GIS model. A different source and a different model would result in a surface with a different structure. Thus the specific results obtained in these analyses are specific to the particular contaminant examined. Nevertheless, the methods used in this paper can be used for any contaminant surface of interest. The generality of the results described here lies in the methods of conducting the kind of the analyses we have described rather than the specific results.

The methods used in this paper can be adapted to other situations where the effect of environmental contaminants on health is the subject of study. Because linked social-spatial data [34-36] increase the risk of identifying the subjects of the research, institutions often limit the quality of the geocoding in order to mask the identity of the subjects. Such masks will severely limit the ability to recover relationships between contaminant exposures and health, especially when the health effects of such contaminants are sensitive to changes in short distances from the sources of exposure [17]. Pursuing such research in rural areas is doubly difficult because the most commonly used spatial mask which moves the location of the respondent from their true location to a masked location is most effective when used in urban areas where the number of other people with whom the respondent could be linked by location is large [35]. Also, large inaccuracies often occur in some geocoding processes in rural areas. It is easier to capture the variability in a contaminant surface in an urban area with the relatively dense settlement pattern of people there.

Our results suggest that studies of relationships between environmental contaminants and health may be better designed by using spatial sampling procedures that identify locations of residences that equalize the number of subjects for different estimated levels of the contaminant load. Random samples of subjects are unlikely to have such characteristics and power analyses based on such samples will be less effective. With the widespread availability in the U.S. and elsewhere of E-911 or similar master address lists, and the availability as in this study of spatially modelled contaminant surfaces, determining such spatially stratified random samples that parsimoniously identify respondent locations will improve the quality of analyses of effects of contaminants on health.

A common problem faced by researchers of this subject is that they cannot know *a priori *whether the quality of the geocoding process they have used is adequate for the purpose of finding a relationship between contaminant values and health. This study is a model of how they might proceed to determine the ability of their proposed research to determine the health effects of the contaminant they are studying by performing the same experiments described in our study. In these experiments they would control the size of sample, the location characteristics of their sample, and the degradation of the geocoding quality of the locations they examine. Some of the studies of geocoding quality include maps of expected geocoding error-rates. These too, when available, can be incorporated in these experiments. We expect that software that automates such experiments will become available in the future. It is needed and could be produced.

Analyses to predict the ability to detect relationships between contaminant values at given locations and health will generally need to incorporate known demographic covariates that are also predictive of a health effect. Power analyses can be designed to incorporate covariates. A recurring question in geographic information science is whether particular geospatial databases are sufficiently accurate for the purpose for which they are used. Determining "fitness-for-use" of a geospatial data set is difficult and has been the subject of research in GIScience [37-42]. An interesting case in point is a study by Lewis et al., [43] which estimated the effect of road traffic exposures to the prevalence of asthma in a sample of 11,562 UK children. The geocode used was the UK postal code which places each child in a relatively large area from the spatial centroid of which the distance to nearest main road was computed. Because of the errors in these estimates of distance from the child's home to the nearest main road, errors in exposure estimates were large, and probably large enough to question the conclusion of the study that asthma prevalence was not associated with proximity of the home to a main road.

Although spatial databases are becoming more accurate as GIS technology improves and efforts are made to improve the accuracy of geographic base maps, it is accepted that no single level of accuracy will meet the requirements of every purpose for which spatial data is used. For each use, there are accuracy requirements and the question we asked is which of three widely used measures of location is adequate for the purpose of assessing whether a relationship exists between exposure to environmental contaminants and health. While research in geocoding accuracy and environmental health problems has often focussed on the effect of inaccuracies on an observed prevalence or relationships [14,44,45], this is to our knowledge the first time the effect of geocoding inaccuracies on assessing the strength of an existing relationship has been addressed. Consideration of such inaccuracies in epidemiologic studies of environmental exposures can greatly improve confidence in the validity and accuracy of results.

## Conclusion

An experimental method to investigate the effect of geocoding accuracy is proposed in this paper. The method of accuracy assessment takes into consideration the 'purpose of use' of the geocodes in an environmental health context. Since a goal of such research is to examine relationships between health and exposure, the proposed method focuses on estimation of disease risk in the presence of modelling errors introduced through geocoding inaccuracies. We examine three widely used geocoding processes. Health data are simulated using known odds from exposure to a contaminant. The contaminant values are calculated using a gold standard geocode. These odds are then detected using contaminant values calculated using two other (apart from the gold standard) geocodes. Of the three geocoding processes studied all were successfully able to recover the simulated odds, though the strength of the relationship varied from process to process. In these analyses E-911 geocoding came out superior to TIGER geocoding (with and without offset). More research is required to decide on an 'optimal geocode', since we have not evaluated all possible offsets of TIGER geocoding, E-911 with offsets and other geocoding processes such as GPS based or parcel based geocoding. Sensitivity analyses show relative robustness of the model at recovering the simulated odds. While the specific results obtained in this research may not be generalized to other situations the method can be applied in any situation where issues of geocoding accuracy are in question in an environmental epidemiological study. Our research extends the literature in geocoding quality analysis by placing it in the context of decision making in environmental epidemiological studies.

## Competing interests

The author(s) declare that they have no competing interests.

## Authors' contributions

SM proposed the experimental design for the paper, conducted the analyses and wrote sections of the paper. GR oversaw the GIS part of the research and wrote sections of the paper. BJS wrote the program for the simulation (in R) which SM modified for this research and extensively reviewed the paper. DZ reviewed statistical details of the paper and revised the statistical sections. KJD directs the research project to which this paper contributes. He wrote sections of the paper.

## References

[B1] Rushton G, Armstrong MP, Gittler J, Greene BR, Pavlik CE, West MM, Zimmerman DL (2006). Geocoding in cancer research: a review. Am J Prev Med.

[B2] Krieger N, Waterman P, Lemieux K, Zierler S, Hogan JW (2001). On the wrong side of the tracts? Evaluating the accuracy of geocoding in public health research. Am J Public Health.

[B3] Zimmerman DL, Fang X, Mazumdar S, Rushton G (2007). Modeling the probability distribution of positional errors incurred by residential address geocoding. Int J Health Geogr.

[B4] Bonner MR, Han D, Nie J, Rogerson P, Vena JE, Freudenheim JL (2003). Positional accuracy of geocoded addresses in epidemiologic research. Epidemiology.

[B5] Carretta HJ, Mick SS (2003). Geocoding public health data. Am J Public Health.

[B6] Cayo MR, Talbot TO (2003). Positional error in automated geocoding of residential addresses. Int J Health Geogr.

[B7] Croner CM (2003). Public health, GIS and the internet. Annu Rev Public Health.

[B8] McElroy JA, Remington PL, Trentham-Dietz A, Robert SA, Newcomb PA (2003). Geocoding addresses from a large population-based study: lessons learned. Epidemiology.

[B9] Oliver MN, Matthews KA, Siadaty M, Hauck FR, Pickle LW (2005). Geographic bias related to geocoding in epidemiologic studies. Int J Health Geogr.

[B10] Ward MH, Nuckols JR, Giglierano J, Bonner MR, Wolter C, Airola M, Mix W, Colt JS, Hartge P (2005). Positional accuracy of two methods of geocoding. Epidemiology.

[B11] Ward MH, Giglierano JB, Wolter C, Miller R, Nuckols JR, Hartge P (2003). How accurately does geocoding determine residential location in rural and urban areas. Epidemiology.

[B12] Gilboa SM, Mendola P, Olshan AF, Harness C, Loomis D, Langlois PH, Savitz DA, Herring AH (2006). Comparison of residential geocoding methods in population-based study of air quality and birth defects. Environ Res.

[B13] Kravets N, Hadden WC (2005). The accuracy of address coding and the effects of coding errors. Health Place.

[B14] Skelly C, Black W, Hearnden M, Eyles R, Weinstein P (2002). Disease surveillance in rural communities is compromised by address geocoding uncertainty: a case study of campylobacteriosis. Aust J Rural Health.

[B15] Krieger N, Chen JT, Waterman PD, Soobader MJ, Subramanian SV, Carson R (2002). Geocoding and monitoring of US socioeconomic inequalities in mortality and cancer incidence: does the choice of area-based measure and geographic level matter ?: The Public Health Disparities Geocoding Project. Am J Epidemiol.

[B16] Rull RP, Ritz B (2003). Historical pesticide exposure in California using pesticide use reports and land-use surveys: an assessment of misclassification error and bias. Environ Health Perspect.

[B17] Armstrong MP, Rushton G, Zimmerman DL (1999). Geographically masking health data to preserve confidentiality. Stat Med.

[B18] McKeown-Eyssen GE, Thomas DC (1985). Sample size determination in case- control studies: the influence of the distribution of exposure. J Chronic Dis.

[B19] Census 2000 Tiger Line Data. http://www.esri.com/data/download/census2000_tigerline/index.html.

[B20] ESRI (2001). ArcGIS- version 9.1.

[B21] Goldberg DW, Wilson JP, Craig A (2007). From text to geographic coordinates. URISA J.

[B22] Zimmerman DL (2007). Estimating the spatial intensity of variation in risk from locations coarsened by incomplete geocoding. Biometrics.

[B23] Avery RC, Wing S, Marshall SW, Schiffman SS (2004). Odor from industrial hog farming operations and mucosal immune function in neighbors. Arch Environ Health.

[B24] Mirabelli MC, Wing S, Marshall SW, Wilcosky TC (2006). Race, poverty, and potential exposure of middle-school students to air emissions from confined swine feeding operations. Environ Health Perspect.

[B25] Schiffman SS, Miller EA, Suggs MS, Graham BG (1995). The effect of environmental odors emanating from commercial swine operations on the mood of nearby residents. Brain Res Bull.

[B26] Schiffman SS, Studwell CE, Landerman LR, Berman K, Sundy JS (2006). Symptomatic effects of exposure to diluted air sampled from a swine confinement atmosphere on healthy human subjects. Environ Health Perspect.

[B27] AERMOD: AMS/EPA Regulatory Model Improvement Committee. http://www.epa.gov/scram001/dispersion_prefrec.htm#aermod.

[B28] The MathWorks (2007). MATLAB R2007a.

[B29] Microsoft Corporation (2004). Microsoft Visual Basic for Applications.

[B30] Asthma Prevalence, Health Care Use and Mortality: United States, 2003–05. http://www.cdc.gov/nchs/products/pubs/pubd/hestats/ashtma03-05/asthma0305.htm.

[B31] Arif AA, Delclos GL, Lee ES, Tortolero SR, Whitehead LW (2003). Prevalence and risk factors of asthma and wheezing among US adults:an analysis of the NHANES III data. Eur Respir J.

[B32] Field RW, Steck DJ, Smith BJ, Brus CP, Fisher EL, Neuberger JS, Platz CE, Robinson RA, Woolson RF, Lynch CF (2000). Residential radon gas exposure and lung cancer: the Iowa Radon Lung Cancer Study. Am J Epidemiol.

[B33] Swine Manure Management and Iowa's Manure Law. http://www.extension.iastate.edu/Publications/PM1700.pdf.

[B34] Guttman MP, Stern PC (2007). Putting people on the map: Protecting confidentiality with linked social-spatial data.

[B35] Sweeney L (2002). K-Anonymity: a model for protecting privacy. Int J Uncertain Fuzz.

[B36] Zimmerman DL, Pavlik C (2008). Quantifying the effects of mask metadata disclosure and multiple releases on the confidentiality of geographically masked health data. Geog Anal.

[B37] Goodchild M (1994). The accuracy of spatial databases.

[B38] Guptill SC, Morrison JL (1995). Elements of Spatial Data Quality.

[B39] Ratcliffe JH (2001). On the accuracy of TIGER-type geocoded address data in relation to cadastral and census areal units. Int J Geog Info Science.

[B40] Whitsel EA, Quibrera PM, Smith RL, Catellier DJ, Liao D, Henley AC, Heiss G (2006). Accuracy of commercial geocoding: assessment and implications. Epidemiol Perspect Innov.

[B41] Whitsel EA, Rose KM, Wood JL, Henley AC, Liao D, Heiss G (2004). Accuracy and repeatability of commercial geocoding. Am J Epidemiol.

[B42] Strickland MJ, Siffel C, Gardner BR, Berzen AK, Correa G (2007). Quantifying geocode location error using GIS methods. Environ Health.

[B43] Lewis SA, Antoniak M, Venn AJ, Davies L, Goodwin A, Salfield N, Britton J, Fogarty AW (2005). Secondhand smoke, dietary fruit intake, road traffic exposures, and the prevalence of asthma: a cross-sectional study in young children. Am J Epidemiol.

[B44] Zandbergen PA (2007). Influence of geocoding quality on environmental exposure assessment of children living near high traffic roads. BMC Public Health.

[B45] Rushton G, Armstrong MP, Gittler J, Greene BR, Pavlik CE, West MM, Zimmerman DL (2007). Geocoding Health Data: The Use of Geographic Codes in Cancer Prevention and Control.

